# Radio-selective effects of a natural occurring muscle-derived dipeptide in A549 and normal cell lines

**DOI:** 10.1038/s41598-019-47944-5

**Published:** 2019-08-08

**Authors:** Norma Ybarra, Jan Seuntjens

**Affiliations:** Cancer Research Program, Research Institute McGill University Health Center, Medical Physics Unit, Gerald Bronfman Department of Oncology, Montreal, H4A 3J1 Canada

**Keywords:** Cancer metabolism, Biological physics

## Abstract

Radiotherapy (RT) causes morbidity and long-term side effects. A challenge in RT is to maximize cancer cells killing while minimizing damage to normal tissue. The ideal radio-protector selectively improves survival and limits damage to normal tissues while reducing survival of cancer cells. Muscle-derived dipeptide, L-carnosine (CAR) is a potent antioxidant, with radio-protective, but also anticancer properties, affecting the cell cycle of cancer cells. We tested CAR effects in lung cancer cells, differentiated and undifferentiated normal cells. We hypothesized that CAR antioxidant properties will confer protection to the two normal cell lines against RT, while preventing lung cancer cell proliferation, and that CAR may act as a radiosensitizer of lung cancer cells due to its effects on cell-cycle progression of cancer cells. Under the experimental conditions reported here, we found that CAR increased radio-sensitivity of lung (A549) cancer cells by increasing the percentage of cells in G2/M (radiosensitive) phase of cell cycle, it negatively affected their bioenergetics, therefore reduced their viability, and DNA-double strand break repair capacity. CAR had either no effect or reduced RT-induced damage in normal cells, depending on the cell type. CAR is a versatile natural occurring compound, that could improve RT-induced lung cancer cells killing, while reducing the damage to normal differentiated and undifferentiated cells.

## Introduction

Cancer is one of the leading causes of mortality and morbidity worldwide (World Health Organization, https://www.who.int/cancer/en/). In addition to surgery and chemotherapy, radiation therapy is an important treatment modality used in cancer treatment. Approximately 50% of all cancer patients will receive radiation therapy during their course of illness^1^, it has been estimated that radiation therapy contributes to around 40% of the curative treatment^2,3^.

Unfortunately, the most prevalent cancer treatments, radiotherapy and chemotherapy, also affect normal tissues, and in both cases the oxidative stress and tissue inflammation caused by these treatments affect targeted and non-targeted cells. The sustained effects of these treatments lead to normal tissue toxicities causing acute and late morbidities (for review see^2,4^).

Cancer patients are treated with different approaches including surgery, RT, and chemotherapy, combining these modalities allows attacking cancer cells by multiple approaches, ideally preventing resistance of cancer cells to each treatment^5^. Often the combination of these treatments exacerbates the deleterious effects in normal tissue and surviving cancer cells could become resistant to these treatments^5^. Chemotherapeutic agents are highly toxic and do not discriminate between normal and cancer cells, these agents often cause severe side effects and could lead to secondary malignancies (for review see^6^).

Radiotherapy (RT) is a more localized treatment, and it is effective to treat the bulky disease, or visible tumour, but it fails to treat the spreading disease^7^. Unfortunately, even though RT is a more localized therapy, it also causes important normal tissue toxicities^2^. A key challenge in RT is to maximize radiation doses to cancer cells while minimizing the damage to surrounding normal tissue^2,8^ and this is achieved, to some extent, by using intensity modulated technologies.

Current cancer therapies have significantly improved cure rates^8^. There is an increase interest in reducing long-term toxicities that are associated with cancer treatments, which negatively affect patients’ quality of life, so strategies aiming to reduce normal tissue toxicities without compromising cancer treatment are imperative^2,8^. The perfect scenario will be to improve current treatment approaches without damaging normal tissues.

Some radio-protectors and drugs have been used to decrease the side effects caused by radiotherapy and chemotherapy, unfortunately with limited success (for review see^9^). Amifostine is a well-known, FDA approved and currently used radio-protector and chemo-protective agent, it selectively protects a broad range of normal tissues (for review see^10,11^). Even though, it has been used with success in the clinic, this compound has side effects, including hypotension, nausea, vomiting and allergic reactions^11,12^. Transient hypocalcemia has been observed when amifostine was administrated in combination with carboplatin and cisplatin^12,13^. On the other hand, knowing that both chemotherapy and radiotherapy perpetuate damage by producing free radicals, multiple vitamin antioxidants and natural compounds have been tested as methods to reduce the toxicity of radiotherapy (for review see^14^) and chemotherapeutic agents, some of these natural compounds such as curcumin and Zingerone have anticancer properties (for review see^15,16^). The major concern with using vitamins and other natural compounds with free-radical scavenging properties is that they are not selective and could also confer free-radical protection to cancer cells^17–19^. The ideal radio-, chemo-protector will be an agent that could selectively improve survival and/or limits the damage to the normal tissue, but at the same time reduces the survival or proliferation of cancer cells.

Several studies report the beneficial effects conferred by L-carnosine (CAR) in many different pathologies. CAR is a cytoplasmic dipeptide synthesized by endogenous carnosine synthetase from β-alanine and histidine and it is found at high concentrations in the skeletal muscle of vertebrates^20^. Several possible roles of this dipeptide have been described, and include pH buffering (for review see^21,22^), metal-ion chelation (for review see^23^), and neurotransmitter function (for review see^24^). Recently it has been described that it has protective effects against oxidative stress and has been coined as a potent antioxidant^25^.

Other interesting beneficial effects attributed to CAR are anti-glycation/transglicating activities^26^ used to prevent and treat age-related and metabolic diseases, such as eye cataracts^27^, prevention of Diabetes mellitus^28^, cardiometabolic risk and disease^29^.

Moreover, several reports describe that CAR has important anticancer properties; these have been documented using *in vivo* and *in vitro* models. *In vitro* studies have shown that CAR inhibited cell growth of human cervical carcinoma cells up to 23%^30^. It also inhibited glioblastoma cells proliferation and this effect was accompanied by an increased expression of manganese superoxide dismutase as well as an increase in cyclin B1 expression, resulting in G2-block^31^.

CAR also decreased human gastric cancer cells proliferation, Shen *et al*.^32^, reported that this effect was mediated by a decrease in the absolute value of mitochondrial ATP-linked respiration, and a reduction of the maximal oxygen consumption and spare respiratory capacity, which may reduce mitochondrial function correlated with proliferative potential. CAR also reduced the extracellular acidification rate and glycolysis^32^. CAR induced apoptosis/cell cycle arrest in human colorectal cancer cells by suppressing of NF-κB/STAT1 signaling^33^.

The *in vivo* anticancer properties of CAR have been described using animal models, Nagai *et al*., first reported a significant decrease of tumour growth in a mouse model of solid tumour sarcoma-180, cancer cells were implanted subcutaneously^34^. In addition, Renner *et al*., reported that CAR delays tumour growth, created by implanting NIH3T3 fibroblasts conditionally expressing the human epidermal growth factor receptor 2 (HER2/neu), using a mouse model. Although CAR was not able to completely prevent tumour growth, the microscopic examination of tumours revealed that those treated with CAR had a significantly lower mitotic index, indicating that CAR reduces the proliferation rate of NIH3T3 fibroblasts conditionally expressing HER2/neu^35^.

Moreover, the radio-protective effects of CAR have been previously reported, a compound of zinc L-carnosine significantly retarded radiation esophagitis in patients with non-small cell lung cancer receiving chemoradiotherapy^36^. CAR also decreased apoptosis and protected testicular seminiferous tubules from gamma-radiation-induced injury in a mouse model^37^.

In the present study, we aim to test the effects of CAR in three different cell types, representing the cell types that are affected by the most currently used therapies to treat cancer, radiotherapy and chemotherapy. Differentiated normal tissues, represented in this case by a primary cell line of human lung fibroblasts; normal undifferentiated cells represented by adherent component of bone marrow cells^38,39^; and cancer cells, represented by the lung cancer cell line A549. We hypothesize that the potent antioxidant and beneficial properties of CAR, previously described will confer protection against radiation to normal differentiated and undifferentiated cells, while preventing lung cancer cells proliferation, and we also hypothesize that CAR could act as a radiosensitizer of lung cancer cells, because it has been reported that CAR blocked cells in G2/M phase^31^, which is the most radiosensitive phase of the cell cycle.

## Results

### Long-term effects of CAR in control and irradiated cells

**Figure 1:**
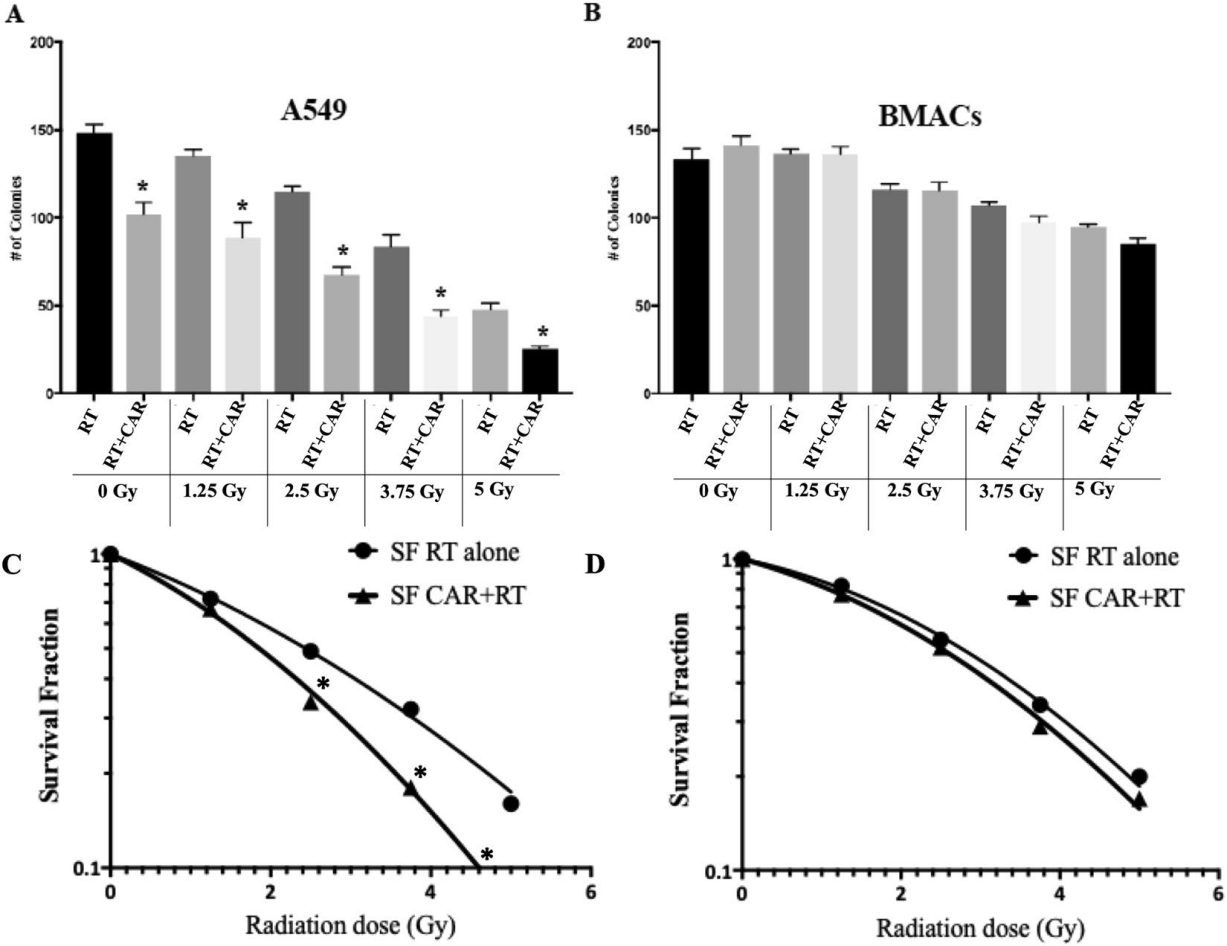
Total number (#) of colonies in A549 cells. (**A**) Bone Marrow adherent cells (BMACs) (**B**) * shows a significant decrease in the number of total colonies counted 10–14 days after radiation, between radiation alone (RT) and pre-treated with CAR and radiated cells (CAR + RT). Survival Fraction (SF) in A549 cells (**C**) BMACs (**D**) * shows a significant decrease of survival fraction between cells treated just with radiation (RT) and the cells pre-treated with CAR and radiated.

Clonogenic cell survival assays were done to assess the long-term effects in cell proliferation and possible radio-sensitization, for A549 cancer cells and bone marrow adherent cells (BMACs), normal cells treated (CAR group, 20 mM) or not (Control group) the day before irradiation. To assess the long-term effects in cell proliferation, cells were allowed to grow in culture for around 10–12 days after irradiation. CAR treatment in the absence of irradiation (Dose 0 Gy) reduced significantly (p < 0.0001) the number of A549 colonies. The reduction of the total number and size of colonies was more evident at increasing radiation doses (Fig. 1A). In the case of BMACs, CAR did not affect the total number of colonies in the absence of radiation (Dose 0 Gy), and when BMACs were irradiated with increasing doses, CAR did not significantly affect the total number of colonies (Fig. 1B). Dose-survival curves were plotted according to the linear quadratic model. The survival fraction of A549 cells pretreated with CAR was significantly lower (p < 0.05) than the survival fraction of A549 cells treated with radiation (RT) alone at doses higher than 2.5 Gy (Fig. 1C), this effect was not observed in BMACs, for which there was no significant difference between cells pretreated with CAR, and the BMACs with RT alone, for all the doses (Fig. 1D). The sensitizer enhancement ratios (SERs) were 1.31 for A549 cells, and 1.08 for BMACs cells.

### Cell cycle progression and DNA double strand breaks

**Figure 2:**
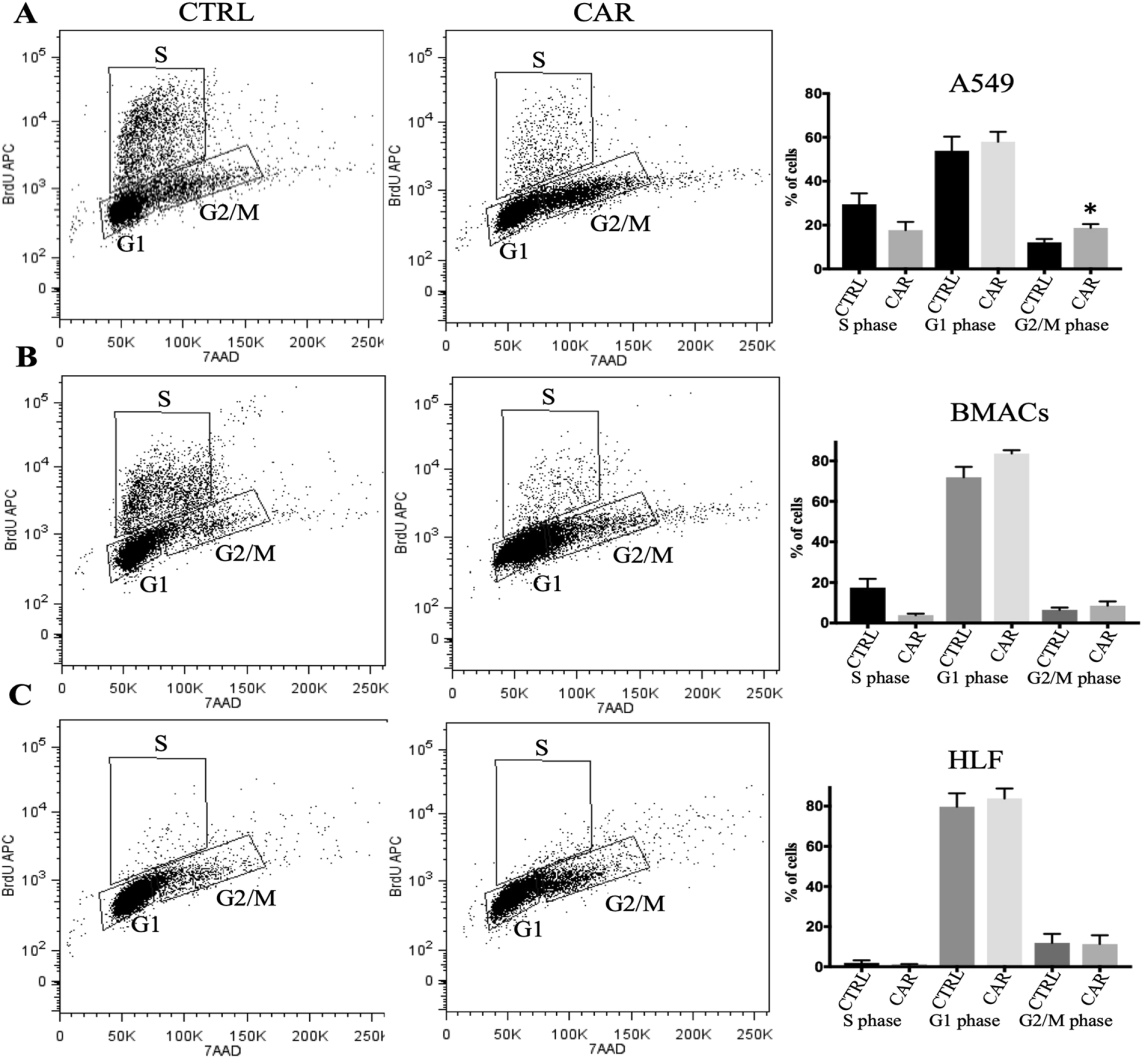
CAR significantly increases the percentage of A549 cells in the G2/M phase of the cell cycle and decreases the percentage of cells in the S phase of the cell cycle for the three cell lines, but this decrease was not significant in any of the three cell lines. Quantification and cell cycle distribution of A549 (**A**), BMACs (**B**), and HLF (**C**) cells were determined by BrdU staining assay and detected by flow cytometry. Cells were treated daily for three days with CAR. Representative images of the flow cytometry analysis of untreated cells (CTRL), left column, and treated cells (CAR) middle column. Data is presented as mean ± S.E.M. (right column) of at least three independent experiments *p < 0.05. Bone marrow adherent cells (BMACs), human lung fibroblasts (HLF).

The effects of CAR in cell proliferation and distribution of cells in the different phases of the cell cycle were determined by flow cytometry. CAR treatment decreased cell proliferation (percentage of cells in S-phase of the cell cycle) of the three cell lines tested, but this decrease was not significant in any of the three cell lines, the cells were treated daily for three days, and analyzed immediately after this period. However, CAR increased significantly the percentage of A549 cells in the G2/M phase (radiosensitive phase); but did not affect significantly the percentage of cells in G2/M phase in the case of the BMACs and human lung fibroblasts (HLF). The percentage of cells in G1, G2/M and S phase for the three cell lines is shown in Fig. 2.

DNA damage was assessed by detecting gamma-H2AX foci. This protein is phosphorylated in response to DNA double strand breaks (DSB). Gamma-H2AX foci were detected and quantified using multispectral imaging flow cytometry. CAR alone increased the percentage of A549 cells having more than 5 foci/nucleus of DNA-DSB, and RT alone further increased this percentage, but the increase was greater and significant when A549 cells were pretreated with CAR and exposed to radiation (2.5 Gy), p = 0.0008, showing an additive effect of CAR. As previously mentioned, CAR alone also increased the percentage of A549 cells locked in the G2/M phase of the cell cycle, possibly making them more radiosensitive. Contrary to the radio-sensitizing effects of CAR in A549 cancer cells, CAR exhibited radio-protective effects to the two normal cell lines tested. When normal cells, BMACs and HLF, were pretreated with CAR and exposed to radiation (2.5 Gy), CAR decreased the percentage of normal cells having more than 5 foci/nucleus of DNA-DSB. The RT alone increased significantly the percentage of BMACs having more than 5 foci/nucleus of DNA-DSB as compared with control. The mean was 53.13 ± 4.34 SEM (p = 0.03), this increase was attenuated, but not statistically significant (RT *vs* CAR + RT). When the BMACs were pretreated with CAR and irradiated, the mean percentage of BMACs having more than 5 foci/nucleus of DNA-DSB for CAR + RT was 35.55 ± 8.18 SEM. There were no statistically significant changes in the HLF among all groups, even though a slight decrease in the percentage of HLF having more than 5 foci/nucleus of DNA-DSB was observed.

**Figure 3:**
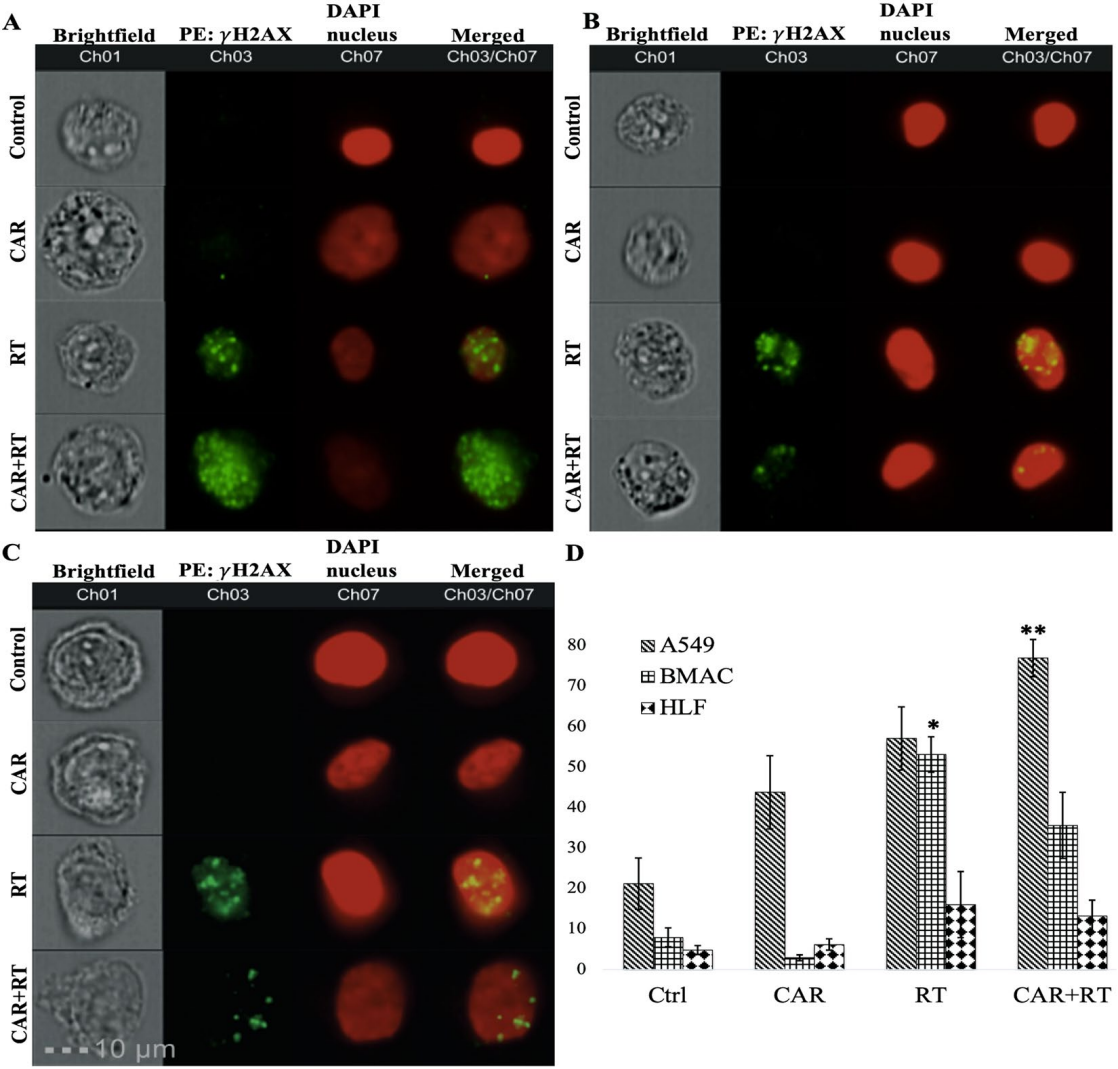
Representative multispectral images of A549 (**A**), BMACs (**B**), and HLF (**C**) cells, untreated, control cells, treated with CAR, RT or both CAR + RT showing γ-H2AX staining for quantification of DNA double strand breaks (DSB) foci. Channel 1 shows a bright-field image; channel 3, γ-H2AX protein foci, channel 7 shows cell nucleus, channel 3/7 represents a merge image. D bar graph shows the percentage of A549, BMACs and HLF having more than 5 foci/nucleus of DNA-DSB. **P < 0.001 compared to control; *P < 0.05 compared to control group.

A summary of the mean percentage ± SEM of the three cell lines having more than 5 foci/nucleus of DNA-DSB, depending on the treatment group is found in Table 1. Representative images of DNA-DSB in the three cell lines and the increase in the percentages of cells having more than 5 foci/nucleus of DNA-DSB for the three cells lines are shown in Fig. 3.

**Table 1:** Mean percentage of the three cell lines having more than 5 foci/nucleus of DNA-DSB, depending on the treatment received.

Cell type	Control mean ± SEM	CAR mean ± SEM	RT mean ± SEM	CAR + RT mean ± SEM
A549	21.17 ± 6.36	43.76 ± 9.06	57.09 ± 7.8	76.93 ± 4.62
BMACs	7.87 ± 2.34	2.88 ± 0.75	53.13 ± 4.34	35.55 ± 8.1
HLF	4.78 ± 1.10	6.12 ± 1.4	16.05 ± 8.1	13.17 ± 3.9

Control cells (CTRL), cells pretreated with CAR (CAR), cells irradiated with a dose of 2.5 Gy (RT), and cells pretreated with CAR and irradiated with a dose of 2.5 Gy (CAR + RT).

### Cell viability and metabolic activity

The XTT assay was used to determine the effects on viability/metabolic activity of cancer and normal cells pretreated with CAR alone and in combination with radiation. The three cell lines were randomized in four groups: the control group, untreated cells; the CAR group, cells treated with CAR (20 mM); the RT group, cells irradiated and untreated with CAR; and the RT + CAR group, cells irradiated and treated with CAR.

**Figure 4:**
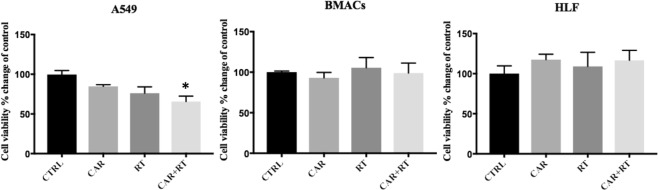
Changes in cell viability/metabolic activity measured using XTT assay. Changes are reported as percentage changes in relation to control cells. Control cells (CTRL), cells pretreated with CAR (CAR), cells irradiated with a dose of 2.5 Gy (RT), and cells pretreated with CAR and irradiated with a dose of 2.5 Gy (CAR + RT). Only A549 cells from group CAR + RT presented a significant decrease in cell viability/metabolic activity p = 0.004. The graphs represent the mean ± SEM of at least three independent experiments carried out in triplicates.

The XTT reduction is also an indicative of cellular metabolic activity. Results showed that when compared to control cells, CAR alone reduced A549 viability/metabolic activity to 81.2%, RT alone reduced it to 85.5% and when CAR pretreated A549 cells were irradiated, cell viability was reduced to 65%, and this reduction was significant p = 0.004 (Fig. 4). Significant changes in cell viability/metabolic activity were not observed in normal cells HLF, and BMACs (Fig. 4).

### Cell bioenergetics

The effects of CAR in cell bioenergetics of the three cell lines were assessed using the Cell mito stress test kit for XF^e^ 96 Extracellular flux analyzer (Seahorse Bioscience, Agilent Technologies). This kit provides the reagents, protocol and analysis method needed to measure the metabolic phenotypes through the quantification of cell oxidative phosphorylation or glycolysis. The test also allows to assess the metabolic potential of living cells; all the parameters and calculations are described in the manufacturer’s protocol (Seahorse Bioscience, Agilent Technologies). Briefly, some of the parameters measured using this technology include; cellular oxygen consumption rate (OCR), which measures oxidative phosphorylation, the extracellular acidification rate (ECAR) which is associated with glycolytic metabolism, it also determines the metabolic potential, which represents the ability of cells to meet an energy demand via respiration and glycolysis. This metabolic potential is calculated as the percentage increase of OCR and ECAR under stressed over baseline conditions. These parameters were simultaneously monitored in the three cell lines which were randomized in four groups: the control group, CAR group, RT group, and RT + CAR group, these groups have been previously described.

**Figure 5:**
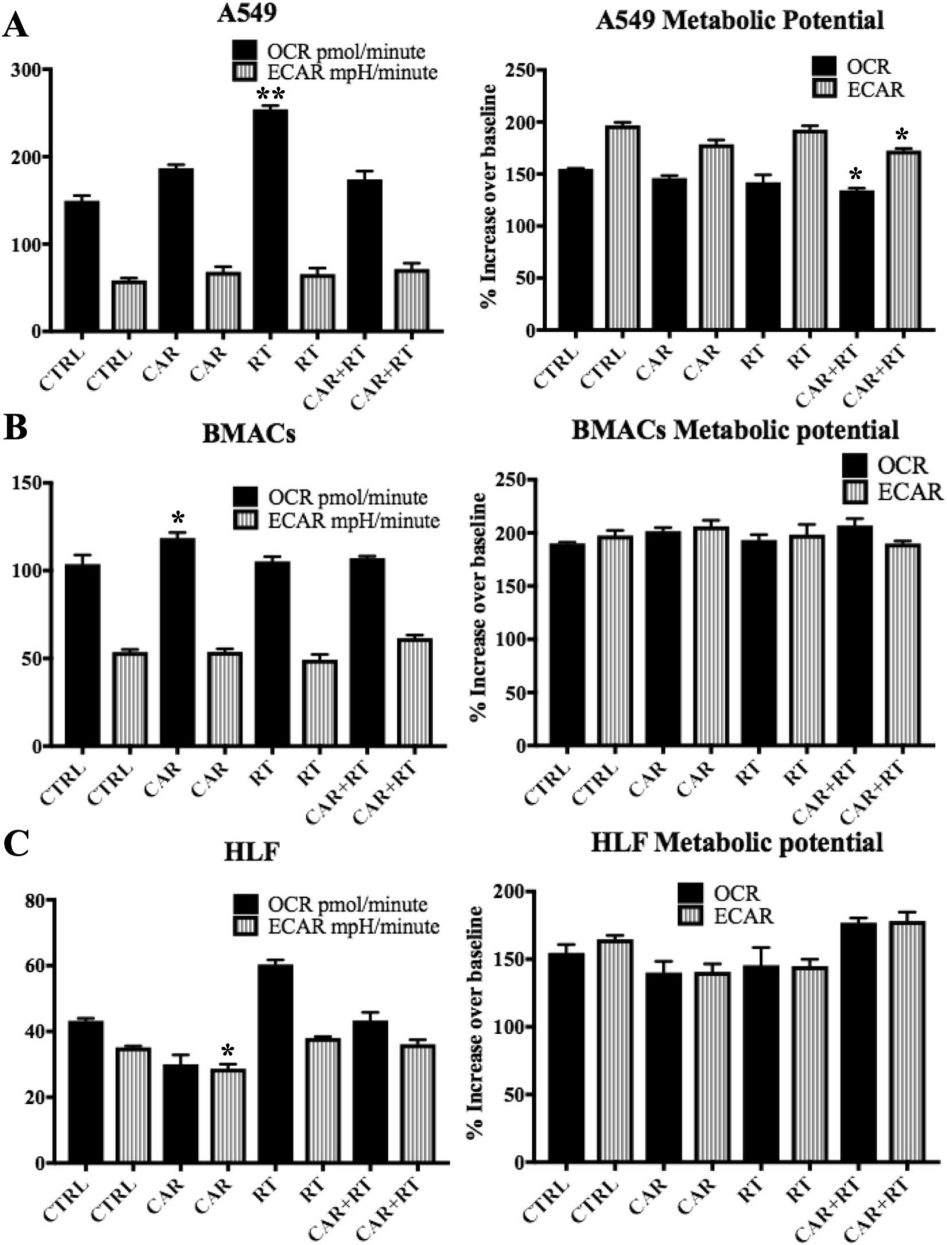
Effects of CAR, RT (2.5 Gy), and CAR + RT on oxygen consumption rate (OCR) resulting from mitochondrial respiration, extracellular acidification rate (ECAR), associated with glycolytic metabolism, and the metabolic potential from respiration (OCR) and glycolysis (ECAR), of A549 (**A**), BMACs (**B**), and HLF cells (**C**). The graphs represent the mean ± SEM of at least three independent experiments carried out in triplicates. *(*p* < 0.05), **(*p* < 0.01) show the significances among treatment groups CAR, RT and CAR + RT compared to control group.

Under baseline conditions the control cells A549 had higher OCR and ECAR values compared to the HLF and BMACs cells. A549 cells had therefore, a higher metabolic activity than that of HLF and BMACs (Fig. 5). The three cell lines from the control group used predominantly oxidative phosphorylation as their main energy producing pathway under baseline conditions. When the cells were subjected to stress, to assess their metabolic potential, we observed that the metabolic potential from glycolysis (ECAR) was higher in A549 cells, than that of the HLF and BMACs. Based on these results we speculate that A549 cells have a higher glycolytic reserve than HLF and BMACs, and that A549 cells relied more in glycolysis than oxidative phosphorylation to meet their energy demands under stress conditions, while HLF and BMACs used the metabolic potential from respiration and glycolysis at similar levels.

In terms of the effects caused by the treatments, an increase in OCR was observed in A549 and HLF cells after irradiation (RT group *vs* control group), but this increase was significant just in A549 cells. CAR treatment attenuated the OCR increase caused by irradiation in A549, and OCR was not significantly different in A549 cells of CAR + RT group when compared to control group. ECAR was not significantly different among treatment groups of A549 cells, when compared to control group. In terms of metabolic potential when compared to control group all treatment groups tend to cause a decrease in the metabolic potential of respiration and glycolysis in A549 cells, but this decrease was significant only for CAR + RT group (Fig. 5A). The changes caused by CAR in normal cell bioenergetics were different than the ones observed in A549 cancer cells. For instance, CAR decreased the OCR and ECAR of HLF, but these parameters were not different in CAR + RT, when compared with the control group. In fact, the metabolic potential from respiration and glycolysis increased in CAR + RT group compared to control group (Fig. 5B), but these increases were not significant. In BMACs, CAR increased OCR compared to control group, but no other significant increases or decreases were observed, when compared to control BMACs (Fig. 5C).

## Discussion

The anti-proliferative effects of CAR in different types of cancer cells have been reported *in vitro* and *in vivo*, and some possible mechanisms have been explored. CAR inhibited carbonic anhydrase IX-mediated extracellular acidosis and suppressed growth of HeLa tumor xenografts^40^. It inhibited proliferation of human gastric cancer cells by decreasing both oxidative phosphorylation and aerobic glycolysis^32^, and its anti-proliferative effect correlated with decreased expression of hypoxia inducible factor-1 alpha (HIF-1α) in human colon cancer cells^41^. However, comparative studies of the effects of drugs, or in this case, a natural occurring compound like CAR, in cancer and normal cells are uncommon in the literature, and most of the time the effects of antineoplastic compounds are solely tested in cancer cells^42–44^, disregarding the possible deleterious effects on normal cells and leading to limited clinical applicability. Comparative studies of the effects of these antineoplastic compounds in normal *vs* cancer cells could help to better understand their mechanism of action, and therefore appropriately explore their potential against cancer cells, by limiting or accurately assessing their possible side effects on normal tissues.

As previously mentioned, CAR is a naturally occurring compound, and in this report the compound was used at concentrations within the physiological ranges. Therefore, it was expected to be innocuous to normal cells. We tested the effects of CAR in two normal primary cell lines and a non-small cell lung cancer cell line (A549 cells), under the same experimental conditions. When we analyzed the short-term effect of CAR on cell proliferation, using the BrDU incorporation assay, we found that CAR decreased cell proliferation in the three cell lines, for this purpose CAR was added daily for three consecutive days.

However, when long-term effects of a single treatment of CAR were tested using the cell clonogenic assay, we observed that it only significantly decreased proliferation of A549 cancer cells, and that this effect was more important when A549 cells were irradiated, indicating a possible radiosensitization effect. CAR did not affect the proliferation of normal BMACs and did not significantly reduce the number of BMACs normal cells colonies. Previous experiments describing the anti-proliferative effects of CAR on cancer cells did not test the possible anti-proliferative or other effects on normal cells. McFarland and Holliday reported the effects of CAR (20–50 mM) in normal fibroblasts (HLF), and fetal lung cells (MRC-5). They reported the effect of CAR on the morphology, growth and longevity of these cells. CAR retarded senescence of late-passage fibroblast cultures and the authors also mentioned that CAR increased the population doubling time, but they did not mention the effects on cell cycle progression or proliferation^45^.

Under the experimental conditions reported here, we observed that CAR seems to pause or delay the cell cycle of the three cell lines tested. However, it affected differently the cell cycle of the two normal cells and A549 cancer cells, even though it decreased the percentage of cells in S phase in the three cell lines, as observed using the cell cycle assay. In the case of the two normal cells tested, CAR increased the percentage of cells in the G1 phase, without affecting the percentage of cells in G2/M phase (radiosensitive). In the case of A549 cells, CAR increased the percentage of cells in the G2/M, consistent with the observation by Rybakova *et al*., who reported that CAR induced G2/M block in cancer cell lines^31^.

There are only few studies reporting some possible mechanisms of the effects of CAR on the cell cycle of cancer cells, and these do not mention if the effects on the cell cycle were the result of adding CAR to the growth culture media at a single time point or at several times during cell culture^31,33,46^. No information is available regarding the effects of CAR on cell cycle progression of normal cells. McFarland *et al*., described an increase in the longevity and population doublings of fibroblasts^45^, but although these described effects could have been mediated by a pausing or a slowing down effect of cell cycle progression caused by CAR, this has not been previously tested or reported by the authors.

The cell cycle is a complex process with high energy demands. However, there is little information on how cells regulate the generation of energy during the different phases of the cell cycle^47^, and it is possible that energy demands, and cell cycle progression are different in cancer cells *versus* normal cells.

A substantial amount of mitochondrial energy is required for cell-cycle progression. The mechanisms underlying the coordination of the mitochondrial respiration with cell cycle progression, especially the G2/M transition, remains to be elucidated^48^. In colon cancer cells, the ATP production in G2/M phase is dependent on mitochondrial respiration, while G1 phase cells rely more on glycolysis for their energy needs during the cell cycle, and the most active mitochondrial oxidative phosphorylation is found to occur in G2/M phase^46^, but these processes seem to differ depending on the cell type^47^. In the present report, we observed that CAR alone slightly increased oxidative phosphorylation in A549 cancer cells compared to control cells. The increase in oxidative phosphorylation caused by CAR could have provided the energy required for having an increased percentage of A549 cancer cells in the G2/M phase, since more ATP was available for cells to progress in these phases of the cell cycle.

Unfortunately, there are only a few reports describing the molecular connections between the generation of energy and cell cycle progression, and these are still very unclear, mainly because of technical limitations and contradictory observations^47^. A clear understanding of energy demands during cell cycle progression in cancer and normal cells could be exploited as an additional cancer therapy, since cancer cells bioenergetics and cell cycle progression are different from that of most normal cells.

Since we observed that CAR selectively increased the percentage of cancer cells in the radiosensitive phase (G2/M) of the cell cycle, we tested the hypothesis that a higher percentage of cancer cells will have a higher number of DNA-DSB. CAR increased significantly the percentage of A549 cancer cells having more than 5 DNA-DSB/cell, but CAR did not have the same effect on the two normal cells tested. Even though, CAR also affected the cell cycle progression of normal cells, it did not affect the radio-sensitivity of normal cells. In fact, the two normal cells treated with CAR had a significantly lower percentage of cells having more than 5 DNA-DSB/cell, when compared with cells exposed to irradiation alone.

Moreover, DNA repair is also a highly energy-demanding process consuming a large amount of cellular ATP^49,50^. Oxidative phosphorylation increases in response to acute and chronic DNA damage^51^, because DNA repair is a highly energy-demanding process, consuming a large amount of cellular ATP^52^. Under the experimental conditions reported here, we observed that radiation increased oxidative phosphorylation in the three cells lines. But when cells were pretreated with CAR and then exposed to radiation, the increase was suppressed in A549 cells and in HLF cells. In addition, we observed a significant decrease in the metabolic potential via respiration and glycolysis only in A549 cancer cells, as previously mentioned the metabolic potential measures the ability of cells to meet an energy demand via respiration and glycolysis, in this case, the energy demand is caused by DNA radiation-induced damage and repair. Surprisingly, the metabolic potential of the two normal cells was not affected by CAR in pretreated cells exposed to RT. CAR did not affect the ability of normal cells to meet energy demands when there is radiation-induced damage and repair, but it did affect the ability of A549 cancer cells to meet energy demands for DNA repair. This could be another reason of why we found a higher percentage of A549 cancer cells having more than 5 DNA-DSB/cell.

Additionally, in order to duplicate genome and biomass, cancer cells have a greater need for energy and an available supply of building blocks required for macromolecules synthesis (nucleotides, protein, lipids)^53^. Usually cancer cells switch their metabolism to aerobic glycolysis, unlike normal cells, which depend on oxidative phosphorylation for energy production. While the exact reasons remain unclear, there are a few possible explanations for this: aerobic glycolysis, although less efficient than the classical oxidative phosphorylation, provides a rapid supply of ATP. In addition, glycolysis intermediates provide sufficient building blocks for macromolecule synthesis required for the enhanced cell proliferation^54^. Lu *et al*., reported that breast cancer MCF-7, colon cancer HCT116 and glioblastoma U87 cancer cell lines switched to oxidative phosphorylation after irradiation and found that this mechanism was mediated via mTOR-Mediated Hexokinase II Inhibition, producing a Warburg-reversing effect. These authors reported a mechanism by which tumor cells can quickly adapt to radiation-induced DNA damage via mTOR-mediated reprogramming of bioenergetics from predominantly aerobic glycolysis to mitochondrial oxidative phosphorylation. The possibility of switching to mitochondrial bioenergetics demonstrates a flexible feature in the energy metabolism of cancer cells and may be required for additional cellular energy consumption for DNA damage repair and cell survival^55^.

CAR was able to decrease significantly the metabolic potential of both oxidative phosphorylation and glycolysis in A549 cancer cells, without affecting normal cells. This shows a selective feature of CAR in inducing a possible radiation sensitivity in A549 cancer cells. The exact mechanism through which CAR selectively affects cancer cell bioenergetics is still unclear, but a better understanding is required to exploit CAR-mediated antineoplastic and possible radiosensitizing properties on A549 cells, at their full potential. Moreover, it will be interesting to test these possible radiosensitizing properties in other cancer cell lines and verify the effects of CAR in a wider range of normal cells as well.

On the other hand, the radio-protective effects of CAR in normal tissues have been previously described. Haeri *et al*., reported that pre-treatment with CAR prevented testicular dysfunction induced by gamma-irradiation, by reducing apoptosis of cells in the seminiferous tubule^37^. Studies reporting the protective effects of CAR against oxidative stress and DNA damage through different mechanisms have been described: CAR attenuated the bone marrow suppression induced by cyclophosphamide in a mice model, by reducing oxidative DNA damage^56^. In a rat animal model pre-treatment with CAR reduced the intestinal damage caused by sodium nitrite, frequently used as a food preservative, which increases oxidative stress in the intestinal epithelium^57^. In addition, it has been reported that an orally bioavailable chelate composed of zinc and L-carnosine reduces oral mucositis^58^, and esophagitis^36^ in cancer patients treated with chemo-radiotherapy.

As reported in the above-mentioned studies, we also observed a decrease in ROS production after irradiation in normal cells pre-treated with CAR, although the effect was not significant (Supplemental Data). The lack of significance could have been related to the limitations of the technique used for ROS detection. ROS measurement in biological systems is problematic and in most of the cases, it is more qualitative than quantitative. All commonly used ROS detection techniques have limitations (for review see^59,60^). On the other hand, we observed an increase of ROS production in A549 pre-treated with CAR and exposed to irradiation, showing again that CAR differentially affects normal and A549 cancer cells. Iovine *et al*., reported that CAR inhibited the proliferation of human HCT116 colon cancer cells and this was mediated by a decrease of ATP and ROS concentration, but they used higher concentrations of CAR (50–100 mM) and they do not mention how long or how they treated the cells with CAR^41^. In our study, CAR was used at physiological concentration (20 mM) and at this concentration, CAR alone did not decrease ROS production, but it did reduce the proliferation of A549 cancer cells. Moreover, it slightly increased ROS production when A549 cells pre-treated with CAR were exposed to radiation compared to radiation alone. Alternatively, CAR reduced or attenuated ROS production in normal cells pre-treated with CAR and exposed to RT, showing an antioxidant effect.

ROS are powerful signaling molecules regulating a variety of biological processes. Cancer cells have higher ROS production than normal cells^61,62^. The higher levels of ROS in cancer cells contribute to the biochemical and molecular changes required for tumor initiation, promotion and progression, as well as for tumor resistance to chemotherapy^62^. On the other hand, most commonly used cancer treatments, including radiation, involve an increase in ROS generation to highly toxic levels that activate ROS-induced cell death pathways. Cancer cells adapt to increased ROS levels by enhancing their antioxidant defense^63^. In normal cells, ROS levels are well controlled by an inducible antioxidant program which responds to cellular stressors. This program is mostly regulated by the transcription factor Nrf2 and its repressor protein Keap1. But there is evidence that cancer cells have increased basal activation of Nrf2^64^. On the other hand, as cancer cells highly rely in ROS signaling, they are also more vulnerable to additional instabilities in their redox status than normal cells. This specific feature of cancer cells could be exploited by not only enhancing ROS production, but also by disturbing their antioxidant defense system, and therefore rendering them more sensitive to therapies which cause cell death by increasing ROS, such as radiation^63^.

Under the experimental settings reported here, CAR increased ROS production exclusively in A549 cancer cells exposed to irradiation, we could speculate that CAR caused an instability in A549 cancer cells redox status causing increased damage to A549 cells, further experiments will be planned to test this hypothesis. In addition, CAR significantly decreased their viability and clonogenicity as tested using XTT assay and clonogenic assay respectively, without affecting these on the two normal cells tested.

In summary, here we report that CAR selectively sensitizes A549 cancer cells to radiation while protecting HLF and BMACs cells. We plan to test the effects observed in this report using other cancer cell types, even though CAR anticancer properties have been extensively documented *in vitro*, we would like to test CAR radio-sensitizing effect in other cancer cell lines, and we are also planning to test a wider range of normal cells. Ultimately, the goal will be to test CAR radio-sensitizing effects in an orthotopic cancer model, a couple of *in vivo* models have previously described CAR anticancer effects in induced tumor models^34,35^. Renner *et al*., described that intraperitoneal injection of CAR (500 μl; 1 M CAR solution), was able to elicit serum concentrations above 20 ± 5 mM, for at least 30 minutes after injection, and that CAR was able to reduce tumor size and mitotic index in this animal model^35^. Even though serum CAR can be hydrolyzed by the enzyme carnosinase, affecting CAR concentrations^65^, the dosing used by Renner *et al*. was able to decrease the tumor size and mitotic index in their animal model.

The mechanisms by which CAR affects differently these two normal cells versus A549 cancer cells seem to be complex and require further investigation. It seems that some of these effects are mediated by differences in the bioenergetics of normal *vs* cancer cells, and also its effects on ROS production. Mitochondrial dysfunction and altered bioenergetics are important characteristics of cancer cells. Here we report the specificity of CAR targeting these two characteristic features of A549 cancer cells.

## Methods

All cell culture reagents, unless stated, were purchased from Thermo Fisher Scientific, Burlington, ON, Canada.

### Cell culture

Primary Lung Fibroblasts, Normal Human (HLF) and lung carcinoma (A549) cell lines were purchased from American Type culture Collection (ATCC Manassas, VA, USA). The HLF cells were cultured in DMEM, high glucose, A549 cells were grown in Ham’s F-12K (Kaighn’s medium), for both cell lines growth culture media were supplemented with 10% fetal bovine serum (FBS) and antibiotic-antimycotic mix. Bone marrow adherent cells (BMACs) were obtained from the femur marrow cavities of Sprague-Dawley rats (Charles River Laboratories, Senneville, QC, CA). This procedure was approved by the Animal Care Committee at the Research Institute of the McGill University Health Centre and in accordance with the ethical guidelines of the Canadian Council on Animal Care. The rats were 7 to 8 weeks old. The femur cavities were exposed under sterile conditions and flushed using a 10 mL syringe attached to 20G needle containing complete growth media DMEM. The fluid was collected in 50 mL sterile tubes and passed through a 70 μm cell strainer. The cells were washed thrice by centrifugation at 400 g for 5 minutes, using complete growth media. The cells were cultured in T-75 culture flasks at 37 °C, 5% CO_2_ in DMEM supplemented with 10% fetal bovine serum (FBS) and antibiotic-antimycotic mix, the medium was changed for the first time after 48 h to eliminate non-adherent cells, and twice a week subsequently. The cells were trypsinized and subcultured when reached 80 to 90% of confluence. Only cells from passage 2–8 were used for these experiments^39^.

The experimental groups for all cells were as follows, Control (Ctrl) cells are cells not treated with CAR and not irradiated, cells treated with CAR but not irradiated are CAR cells, cells irradiated but not treated with CAR are RT cells, and cells treated with CAR and irradiated are CAR + RT cells.

### Clonogenic assay

Clonogenic assay was used to test the long-term effects of radiation at different doses, on the A549 and BMACs cell lines in CAR treated cells or non-treated control cells. In the case of HLF clonogenic assays were also done to test the effect of different doses of radiation in CAR treated and control cells (Sigma-Aldrich, Oakville, ON, Canada), but this type of cells did not exhibit well-formed colonies, and it was difficult to interpret data obtained from these clonogenic assays, therefore data from HLF is not shown in the present report, and other methods to test proliferation were used. Only Colonies with 50 or more cells were counted.

A549 and BMACs cells were cultured in 6-well plates at optimized cell seeding densities, depending on the cell type, and irradiated at 0, 1.25, 2.5, 3.75 and 5 Gy (Faxitron x-ray Corporation). Increasing number of cells was used for increasing doses of radiation. CAR was added to the media, the day before irradiation at a concentration of 20 mM, this concentration was found to inhibit proliferation of transformed and neoplastic cells^66^.

At 10–12 days after irradiation, growth media was removed, and cells were washed with PBS and fixed in 10% formalin (Sigma-Aldrich, ON, Canada) for two hours, and stained with 0.5% Methylene blue (Sigma-Aldrich, ON, Canada) in PBS overnight, the plates were washed with running water, and left to dry overnight. Stained colonies were counted, only the colonies containing more than 50 individual cells were considered in the counting. Cells were counted using bright field microscopy (Zeiss, Canada).

### Cell viability

Cell viability (metabolic activity) was detected using a commercially available colorimetric XTT assay, following manufacture’s protocol (XTT Cell Viability Kit, Biotium, CA, USA). Briefly, cells were cultured on 96-well plates, at a concentration of 15,000 cells in 100 µL, per well. The cells were treated or not with CAR for three days before irradiation, unirradiated cells were used as controls. Cells were irradiated and two hours after irradiation 25 μl XTT solution was added to control and irradiated cells, plates were incubated for 2 hours after XTT addition. The cell viability was determined by measuring the absorbance of the wells at a wavelength of 450 nm, background absorbance was measured at a wavelength of 630 nm. Background absorbance was subtracted from signal absorbance to obtain normalized values. Results were expressed as the percentage of XTT reduction, taking the absorbance of control cells as 100%.

### Cell proliferation and cell cycle assay

To study the short-term effect of repeated (3x) treatments of CAR in cell proliferation, and to test if it affects the cell cycle of the three different cell types (A549, HLF and BMACs) the cells were cultured in the absence, control cells or the presence of CAR. The cells were treated immediately after plating and media was changed every 24 h. Cells were collected 72 h after first plating. The day of cell collection, BrDU at a concentration of 10 μM was added to the growth media and cells were incubated at 37 °C, 5% CO_2_ for three hours, after this period cells were washed in DPBS and trypsinized with 0.25% Trypsin/EDTA, washed in DPBS, fixed and stained for cell cycle analysis following the protocol described for the APC BrDU flow kit (BD Pharmingen, Toronto ON, CA). Samples data acquisition was done using BD FACSCanto II (BD Pharmingen, Toronto ON, CA) and cell cycle analysis was done using FlowJo Analysis Software version 9, following manufacturer’s instructions.

### Analysis of DNA damage

To test the effects of CAR on DNA damage, DNA double strand breaks were measured by detecting gamma-H2AX foci, using multispectral imaging flow cytometry. The three different cell lines were treated or not with CAR for three consecutive days. After this period, cells were irradiated at a dose of 2.5 Gy (Faxitron x-ray Corporation), un-irradiated cells were used as controls. After irradiation cells were incubated for two hours at 37 °C, 5% CO_2_. After this period, cells were detached by trypsinization (0.25% trypsin/EDTA) and washed twice in phosphate buffered saline (PBS), fixed and stained for intranuclear staining following the protocol of the commercially available kit eBioscience™ Foxp3/Transcription Factor Fixation/Permeabilization, with a few modifications (ThermoFisher Scientific, CA). After fixation/permeabilization as indicated in manufacturer’s protocol, cells were resuspended in blocking buffer, consisting in 10% goat serum in 1x permeabilization buffer, cells were incubated overnight at 4 °C. After which, cells were spun at 400 g for 5 minutes, supernatant discarded. Cells were incubated at 4 °C overnight in mouse monoclonal antiserine139 γ-H2AX antibody, clone JBW 301 (Millipore, CA) at 1:1000 dilution. Cells were washed thrice with 1x permeabilization buffer and re-suspended in anti-mouse IgG (whole molecule) R-phycoerythrin conjugated antibody diluted to 1:50 in blocking buffer (5% goat serum in 1x permeabilization buffer). Cells were incubated for 1 hr at room temperature, washed three times; nuclear staining was done by resuspending cells in DAPI/PBS, at a concentration of 1:1000, before the last wash. Cells were resuspended in flow cytometry staining buffer (2% FBS in PBS). Samples for fluorescence compensation were prepared in which either the secondary antibody (R-phycoerythrin conjugate) or DAPI was omitted from the procedure.

During imaging flow cytometry, images of 10,000 cells were acquired at 40× magnification with and without Extended Depth of Field using the 488 nm excitation laser set at 100 mW. For compensation samples images of 500 cells were acquired illuminated by the 488 nm laser with the brightfield and darkfield lasers inactivated.

Imaging flow cytometry was conducted using Imagestream^x^ system (Aminis Inc., Washington, USA). Gamma‐H2AX foci were quantified as previously described by Bourton *et al*.^67^. Briefly, using the Inspire^TM^ data software, images of 20,000 cells were captured on channel 1 for brightfield (BF), on channel 3 for phycoerythrin (PE), and channel 7 for 4′,6-diamidino-2-phenylindole (DAPI). All images were captured using a 40X objective.

Image compensation was done using irradiated cells, which were previously fixed and stained; PE staining intensity was the highest on these cells. Cells that were stained only with anti-γ-H2AX-PE or DAPI were used for generating the compensation matrix removing the BF illumination. Image analysis was done using the software Ideas^TM^. To determine the number of γ-H2AX foci within the nucleus of the cells analyzed, a series of masks were created as described by Bourton *et al*.^67^, a nuclear mask was created using the DAPI (channel 7) image of each cell. To accurately determine the number of γ-H2AX foci within each cell, a population of cells was identified using the image gallery, images with a manually quantified number of PE foci were identified, the number of foci ranged from zero to 30 or more foci. Using this population, a spot mask was created using the spot function of the mask manager tool. Finally, to determine the number of foci within the nuclear specific region of each cell, a combined mask was created.

### Effects on mitochondrial function

Mitochondrial function was assessed by measuring oxygen consumption rate (OCR) and extracellular acidification rate (ECAR) on live cells (each cell line) using the XF^e^ 96 Extracellular flux analyzer (Seahorse Bioscience, Agilent Technologies) using the Cell mito stress test kit. Cancer and normal cell lines were treated or not with CAR, the day before the assay the cells were trypsinized and seeded at 1.5 × 10^4^ cells/well in a 96 well plate and grown for 24 h. Then cells were washed twice with XF base medium supplemented with 2 mM glutamine, 10 mM glucose, and 1 mM sodium pyruvate, and pH was adjusted to 7.4. Cells were incubated in this medium for 1 h at 37 °C in a non-CO_2_ incubator. The cell mito stress test kit assay protocol was performed as suggested by the manufacturer (Seahorse Bioscience, Agilent Technologies). Oxygen consumption rate (OCR) was detected under basal conditions followed by the sequential addition of oligomycin 1 µm for all cells, Carbonyl cyanide-p-trifluoromethoxyphenylhydrazone (FCCP) 1 µm for BMACs, and 0.5 µm for A549 and HLF, and rotenone/antimycin 0.5 μM for all cells. This allowed for measurement of the following parameters: basal respiration, OCR, ATP production, maximal respiration, spare respiratory capacity, and non-mitochondrial respiration, as described in the protocol provided by the manufacturer (Seahorse Bioscience, Agilent Technologies).

### Statistical analysis

All experiments were performed at least three times and data reported as mean ± S.E.M. Statistical analyses of data were performed using GraphPad Prism software (GraphPad Software, Inc., CA, USA). Difference among different treatments groups was checked for statistical significance using the Kruskal-Wallis test, followed by Dunn’s multiple comparisons test. Differences between two groups, when only control and CAR groups were compared, were calculated by Mann Whitney U test, *p* < 0.05 was considered statistically significant.

## Supplementary information

Dataset 1

## Data Availability

The data generated during and analysed during the current study is included in the manuscript, if further information is required it will be available from the corresponding author upon request.

## References

[1] Delaney G, Jacob S, Featherstone C, Barton M (2005). The role of radiotherapy in cancer treatment. Cancer.

[2] Barnett GC (2009). Normal tissue reactions to radiotherapy: towards tailoring treatment dose by genotype. Nature Reviews Cancer.

[3] Baskar R, Lee KA, Yeo R, Yeoh K-W (2012). Cancer and Radiation Therapy: Current Advances and Future Directions. International Journal of Medical Sciences.

[4] Chen Y, Jungsuwadee P, Vore M, Butterfield DA, Clair DKS (2007). Collateral Damage in Cancer Chemotherapy: Oxidative Stress in Nontargeted Tissues. Molecular Interventions.

[5] Bentzen SM, Harari PM, Bernier J (2007). Exploitable mechanisms for combining drugs with radiation: concepts, achievements and future directions. Nature Clinical Practice Oncology.

[6] Vega-Stromberg T (2003). Chemotherapy-induced Secondary Malignancies. Journal of Infusion Nursing.

[7] Prasanna PG (2012). Normal tissue protection for improving radiotherapy: Where are the Gaps?. Transl Cancer Res..

[8] Herrera FG, Bourhis J, Coukos G (2016). Radiotherapy combination opportunities leveraging immunity for the next oncology practice. CA: A Cancer Journal for Clinicians.

[9] Citrin D (2010). Radioprotectors and Mitigators of Radiation-Induced Normal Tissue Injury. The Oncologist.

[10] Kouvaris JR, Kouloulias VE, Vlahos LJ (2007). Amifostine: The First Selective-Target and Broad-Spectrum Radioprotector. The Oncologist.

[11] Koukourakis M (2002). Amifostine in clinical oncology: current use and future applications. Anti-Cancer Drugs.

[12] Bukowski, R. Amifostine (Ethyol®): Dosing, administration and patient management guidelines. *European Journal of Cancer* **32** (1996). 10.1016/s0959-8049(96)00328-0 8976823

[13] Rades D (2004). Serious adverse effects of amifostine during radiotherapy in head and neck cancer patients. Radiotherapy and Oncology.

[14] Borek Carmia (2004). Antioxidants and Radiation Therapy. The Journal of Nutrition.

[15] Block KI (2008). Impact of antioxidant supplementation on chemotherapeutic toxicity: A systematic review of the evidence from randomized controlled trials. International Journal of Cancer.

[16] Mun G-I, Kim S, Choi E, Kim CS, Lee Y-S (2018). Pharmacology of natural radioprotectors. Archives of Pharmacal Research.

[17] Bairati I (2005). Randomized Trial of Antioxidant Vitamins to Prevent Acute Adverse Effects of Radiation Therapy in Head and Neck Cancer Patients. Journal of Clinical Oncology.

[18] Witenberg B (1999). Ascorbic acid inhibits apoptosis induced by X irradiation in HL60 Myeloid Leukemia Cells. Radiation Research.

[19] Wiernik PH (1992). Hexamethylmelamine and low or moderate dose cisplatin with or without pyridoxine for treatment of advanced ovarian carcinoma: A study of the Eastern Cooperative Oncology Group. Cancer Investigation.

[20] Sale C, Saunders B, Harris RC (2009). Effect of beta-alanine supplementation on muscle carnosine concentrations and exercise performance. Amino Acids.

[21] Smith ECB (1938). The buffering of muscle in rigor; protein, phosphate and carnosine. The Journal of Physiology.

[22] Davey C (1960). The significance of carnosine and anserine in striated skeletal muscle. Archives of Biochemistry and Biophysics.

[23] Brown CE (1981). Interactions among carnosine, anserine, ophidine and copper in biochemical adaptation. Journal of Theoretical Biology.

[24] Snyder S (1980). Brain peptides as neurotransmitters. Science.

[25] Fontana M, Pinnen F, Lucente G, Pecci L (2002). Prevention of peroxynitrite-dependent damage by carnosine and related sulphonamido pseudodipeptides. Cellular and Molecular Life Sciences (CMLS).

[26] Hipkiss AR, Michaelis J, Syrris P (1995). Non-enzymatic glycosylation of the dipeptide l-carnosine, a potential anti-protein-cross-linking agent. FEBS Letters.

[27] Abdelkader H, Longman M, Alany RG, Pierscionek B (2016). On the Anticataractogenic Effects of L-Carnosine: Is It Best Described as an Antioxidant, Metal-Chelating Agent or Glycation Inhibitor?. Oxidative Medicine and Cellular Longevity.

[28] Courten BD (2016). Effects of carnosine supplementation on glucose metabolism: Pilot clinical trial. Obesity.

[29] Baye E (2016). Physiological and therapeutic effects of carnosine on cardiometabolic risk and disease. Amino Acids.

[30] Pandurangan M, Enkhtaivan G, Kim DH (2016). Therapeutic efficacy of natural dipeptide carnosine against human cervical carcinoma cells. Journal of Molecular Recognition.

[31] Rybakova YS (2015). Increased manganese superoxide dismutase and cyclin B1 expression in carnosine-induced inhibition of glioblastoma cell proliferation. Biochemistry (Moscow) Supplement Series B: Biomedical Chemistry.

[32] Shen Yao, Yang Jianbo, Li Juan, Shi Xiaojie, Ouyang Li, Tian Yueyang, Lu Jianxin (2014). Carnosine Inhibits the Proliferation of Human Gastric Cancer SGC-7901 Cells through Both of the Mitochondrial Respiration and Glycolysis Pathways. PLoS ONE.

[33] Lee J (2018). l-carnosine induces apoptosis/cell cycle arrest via suppression of NF-κB/STAT1 pathway in HCT116 colorectal cancer cells. In Vitro Cellular & Developmental Biology - Animal.

[34] Nagai, K. & Suda, T. Antineoplastic effects of carnosine and beta-alanine–physiological considerations of its antineoplastic effects. *Nihon Seirigaku Zasshi* **48**, 741–747 (986AD). 3102721

[35] Renner C (2010). Carnosine retards tumor growth *in vivo* in an NIH3T3-HER2/neu mouse model. Molecular Cancer.

[36] Yanase K (2015). Prevention of radiation esophagitis by polaprezinc (zinc L-carnosine) in patients with non-small cell lung cancer who received chemoradiotherapy. Int. J. Clin. Exp. Med.

[37] Haeri SA, Rajabi H, Fazelipour S, Hosseinimehr SJ (2013). Carnosine mitigates apoptosis and protects testicular seminiferous tubules from gamma-radiation-induced injury in mice. Andrologia.

[38] Bianco P, Robey PG (2000). Marrow stromal stem cells. Journal of Clinical Investigation.

[39] Fang L, Du M, Li Y, Zhang C, Zhao Z (2016). Using the differential adhesion method to isolate and culture mesenchymal stem cells and endothelial progenitor cells from rat bone marrow. Biomedical Research.

[40] Ditte, Z. *et al*. Carnosine inhibits carbonic anhydrase IX-mediated extracellular acidosis and suppresses growth of HeLa tumor xenografts. *BMC Cancer* **14** (2014). 10.1186/1471-2407-14-358 PMC4061103 24886661

[41] Iovine B, Iannella ML, Nocella F, Pricolo MR, Bevilacqua MA (2012). Carnosine inhibits KRAS-mediated HCT116 proliferation by affecting ATP and ROS production. Cancer Letters.

[42] Shin M (2018). Triterpenoids from Ziziphus jujuba induce apoptotic cell death in human cancer cells through mitochondrial reactive oxygen species production. Food & Function.

[43] Piska K, Gunia-Krzyżak A, Koczurkiewicz P, Wójcik-Pszczoła K, Pękala E (2018). Piperlongumine (piplartine) as a lead compound for anticancer agents – Synthesis and properties of analogues: A mini-review. European Journal of Medicinal Chemistry.

[44] Imran M (2018). Thymoquinone: A novel strategy to combat cancer: A review. Biomedicine & Pharmacotherapy.

[45] Mcfarland G, Holliday R (1994). Retardation of the Senescence of Cultured Human Diploid Fibroblasts by Carnosine. Experimental Cell Research.

[46] Bao Y (2016). Carnosine Inhibits the Proliferation of Human Cervical Gland Carcinoma Cells Through Inhibiting Both Mitochondrial Bioenergetics and Glycolysis Pathways and Retarding Cell Cycle Progression. Integrative Cancer Therapies.

[47] Salazar-Roa M, Malumbres M (2017). Fueling the Cell Division Cycle. Trends in Cell Biology.

[48] Wang Z (2014). Cyclin B1/Cdk1 Coordinates Mitochondrial Respiration for Cell-Cycle G2/M Progression. Developmental Cell.

[49] Hořejší Z (2004). Distinct functional domains of Nbs1 modulate the timing and magnitude of ATM activation after low doses of ionizing radiation. Oncogene.

[50] Paull TT, Gellert M (1999). Nbs1 potentiates ATP-driven DNA unwinding and endonuclease cleavage by the Mre11/Rad50 complex. Genes & Development.

[51] Brace, L. E. *et al*. Increased oxidative phosphorylation in response to acute and chronic DNA damage. *npj Aging and Mechanisms of Disease* **2** (2016). 10.1038/npjamd.2016.22 PMC5514997 28721274

[52] Qin L (2015). CDK1 Enhances Mitochondrial Bioenergetics for Radiation-Induced DNA Repair. Cell Reports.

[53] Antone PD (2012). Energy metabolism in cancer cells: How to explain the Warburg and Crabtree effects?. Medical Hypotheses.

[54] Sreedhar, A. & Zhao, Y. Dysregulated metabolic enzymes and metabolic reprogramming in cancer cells (Review). *Biomedical Reports*, 10.3892/br.2017.1022 (2017). 10.3892/br.2017.1022 PMC5772474 29399334

[55] Lu Chung-Ling, Qin Lili, Liu Hsin-Chen, Candas Demet, Fan Ming, Li Jian Jian (2015). Tumor Cells Switch to Mitochondrial Oxidative Phosphorylation under Radiation via mTOR-Mediated Hexokinase II Inhibition - A Warburg-Reversing Effect. PLOS ONE.

[56] Deng J (2018). Carnosine attenuates cyclophosphamide-induced bone marrow suppression by reducing oxidative DNA damage. Redox Biology.

[57] Ansari FA, Khan AA, Mahmood R (2018). Protective effect of carnosine and N-acetylcysteine against sodium nitrite-induced oxidative stress and DNA damage in rat intestine. Environmental Science and Pollution Research.

[58] Suzuki A (2016). Effect of polaprezinc on oral mucositis, irradiation period, and time to discharge in patients with head and neck cancer. Head & Neck.

[59] Ameziane-El-Hassani, R. & Dupuy, C. Detection of Intracellular Reactive Oxygen Species (CM-H2DCFDA). *Bio-Protocol* **3** (2013).

[60] Roesslein M, Hirsch C, Kaiser J-P, Krug H, Wick P (2013). Comparability of *in Vitro* Tests for Bioactive Nanoparticles: A Common Assay to Detect Reactive Oxygen Species as an Example. International Journal of Molecular Sciences.

[61] Wang J, Yi J (2008). Cancer cell killing via ROS: To increase or decrease, that is the question. Cancer Biology & Therapy.

[62] Schumacker PT (2006). Reactive oxygen species in cancer cells: Live by the sword, die by the sword. Cancer Cell.

[63] Sznarkowska, A., Kostecka, A., Meller, K. & Bielawski, K. P. Inhibition of cancer antioxidant defense by natural compounds. *Oncotarget* **8** (2016). 10.18632/oncotarget.13723 PMC5362541 27911871

[64] DeNicola GM (2011). Oncogene-induced Nrf2 transcription promotes ROS detoxification and tumorigenesis. Nature.

[65] Raschke S, Eckel J (2013). Adipo-Myokines: Two Sides of the Same Coin—Mediators of Inflammation and Mediators of Exercise. Mediators of Inflammation.

[66] Holliday R, Mcfarland G (1996). Inhibition of the growth of transformed and neoplastic cells by the dipeptide carnosine. British Journal of Cancer.

[67] Bourton EC (2011). Multispectral imaging flow cytometry reveals distinct frequencies of γ-H2AX foci induction in DNA double strand break repair defective human cell lines. Cytometry Part A.

