# Supplementary material for: Radio-selective effects of a natural occurring muscle-derived dipeptide in A549 and normal cell lines
Source: Sci Rep. 2019 Aug 8;9:11513. doi: 10.1038/s41598-019-47944-5 (PMC6687720; doi:10.1038/s41598-019-47944-5)
Supplement: Supplementary file 1 — Dataset 1 [file 41598_2019_47944_MOESM1_ESM.pdf]

Radio-selective effects of a natural occurring muscle-derived dipeptide in A549 and normal cell lines.

Norma Ybarra*, Jan Seuntjens

Research Institute McGill University Health Center, Department of Oncology, Medical Physics Unit, Montreal, H4A 3J1 Canada

Norma Ybarra Email: norma.ybarra@mcgill.ca
*Corresponding author

Supplemental data

Results modulation of redox balance

To evaluate the induction of oxidative stress and ROS formation the probe CM-H_2DCFDA was used. This membrane permeable probe becomes fluorescent upon oxidation. It is well known that RT elicits the production of ROS. Therefore, as expected, a significant increase in ROS production was observed in the three cell lines, from the RT group in comparison with the control group. When RT and CAR+RT groups were compared, co-treatment with CAR increased ROS production only in A549 cells, and decreased the ROS production in normal cell lines, but this decrease was marginally significant for both normal cells p=0.05. The results of ROS production are expressed as the percentage of H_2DCFDA fluorescence, taking the fluorescence of control cells as 100% (Figure 6).

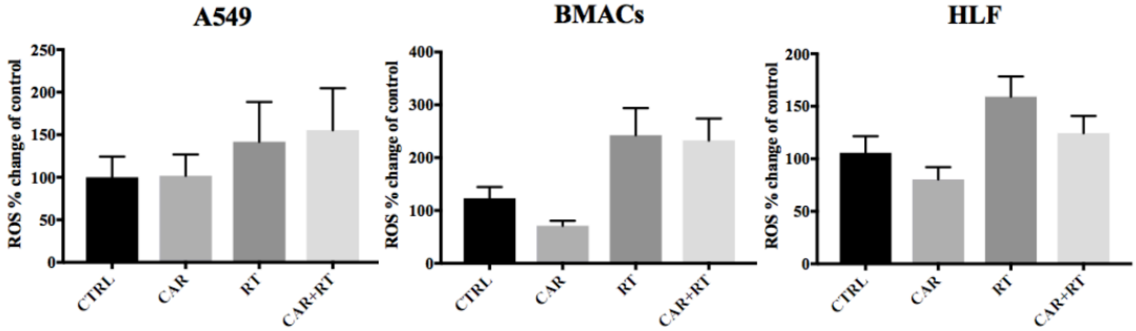

Figure 6. Changes in ROS production using the fluorescent probe CM-H_2DCFDA. Changes are reported as percentage changes relative to control cells. Control cells (CTRL), cells pretreated with CAR (CAR), cells irradiated with a dose of 2.5 Gy (RT), and cells pretreated with CAR and irradiated with a dose of 2.5 Gy (CAR+RT). The graphs represent the mean ± SEM of at least three independent experiments carried out in triplicates. A trend of increased ROS production was observed in all cells from RT group, and this trend was higher only in A549 cells from CAR+RT group, but was not significant. In normal cells, BMACs and HLF, ROS production was lower in CAR group compared to control group, and in CAR+RT group compared to RT alone group, but not significant.

Methods modulation of redox balance

Reactive oxygen species production was measured in treatment groups using ROS-sensitive fluorescent probe 5-(and-6)-chloromethyl-2′,7′-dichloro-di-hydro-fluorescein diacetate, acetyl ester (CM-H_2DCFDA) (Invitrogen, Life Technologies Ltd). Briefly, all cell lines were seeded in 96 well black plates with clear bottom (1.5×10^4 cells/well), media was removed and cells were washed twice with HBSS and incubated with CM-H_2DCFDA (10 μM) for 30 min at 37 °C in the dark, ROS production was detected after

irradiation, as an increase in fluorescence, using a fluorescence microplate reader Infinite M200Pro Tecan (TECAN Group LTD.), at 485 nm excitation and at 535 nm emission.
